# Robust prediction of hourly PM_2.5_ from meteorological data using LightGBM

**DOI:** 10.1093/nsr/nwaa307

**Published:** 2021-01-05

**Authors:** Junting Zhong, Xiaoye Zhang, Ke Gui, Yaqiang Wang, Huizheng Che, Xiaojing Shen, Lei Zhang, Yangmei Zhang, Junying Sun, Wenjie Zhang

**Affiliations:** State Key Laboratory of Severe Weather and Key Laboratory of Atmospheric Chemistry of China Meteorological Administration, Chinese Academy of Meteorological Sciences, Beijing 100081, China; School of Earth and Planetary Sciences, University of Chinese Academy of Sciences, Beijing 100049, China; State Key Laboratory of Severe Weather and Key Laboratory of Atmospheric Chemistry of China Meteorological Administration, Chinese Academy of Meteorological Sciences, Beijing 100081, China; Center for Excellence in Regional Atmospheric Environment, Institute of Urban Environment, Chinese Academy of Sciences, Xiamen 361021, China; State Key Laboratory of Severe Weather and Key Laboratory of Atmospheric Chemistry of China Meteorological Administration, Chinese Academy of Meteorological Sciences, Beijing 100081, China; State Key Laboratory of Severe Weather and Key Laboratory of Atmospheric Chemistry of China Meteorological Administration, Chinese Academy of Meteorological Sciences, Beijing 100081, China; State Key Laboratory of Severe Weather and Key Laboratory of Atmospheric Chemistry of China Meteorological Administration, Chinese Academy of Meteorological Sciences, Beijing 100081, China; State Key Laboratory of Severe Weather and Key Laboratory of Atmospheric Chemistry of China Meteorological Administration, Chinese Academy of Meteorological Sciences, Beijing 100081, China; State Key Laboratory of Severe Weather and Key Laboratory of Atmospheric Chemistry of China Meteorological Administration, Chinese Academy of Meteorological Sciences, Beijing 100081, China; State Key Laboratory of Severe Weather and Key Laboratory of Atmospheric Chemistry of China Meteorological Administration, Chinese Academy of Meteorological Sciences, Beijing 100081, China; State Key Laboratory of Severe Weather and Key Laboratory of Atmospheric Chemistry of China Meteorological Administration, Chinese Academy of Meteorological Sciences, Beijing 100081, China; State Key Laboratory of Severe Weather and Key Laboratory of Atmospheric Chemistry of China Meteorological Administration, Chinese Academy of Meteorological Sciences, Beijing 100081, China

**Keywords:** PM_2.5_, spatial features, hourly prediction, high accuracy, gridded networks

## Abstract

Retrieving historical fine particulate matter (PM_2.5_) data is key for evaluating the long-term impacts of PM_2.5_ on the environment, human health and climate change. Satellite-based aerosol optical depth has been used to estimate PM_2.5_, but estimations have largely been undermined by massive missing values, low sampling frequency and weak predictive capability. Here, using a novel feature engineering approach to incorporate spatial effects from meteorological data, we developed a robust LightGBM model that predicts PM_2.5_ at an unprecedented predictive capacity on hourly (R^2 ^= 0.75), daily (R^2 ^= 0.84), monthly (R^2 ^= 0.88) and annual (R^2 ^= 0.87) timescales. By taking advantage of spatial features, our model can also construct hourly gridded networks of PM_2.5_. This capability would be further enhanced if meteorological observations from regional stations were incorporated. Our results show that this model has great potential in reconstructing historical PM_2.5_ datasets and real-time gridded networks at high spatial-temporal resolutions. The resulting datasets can be assimilated into models to produce long-term re-analysis that incorporates interactions between aerosols and physical processes.

## Teaser

A high-performance machine-learning model incorporating spatial effects was developed to estimate historical PM2.5 concentrations based on meteorological data. Capable of hourly resolution, this dataset will be of great value for understanding PM2.5's long-term climate and environmental effects and producing chemical-weather coupled reanalysis.

## INTRODUCTION

Fine particulate matter (PM_2.5_) consists of suspended and inhalable particles that generate environmental and health effects [1–7]. Suspended PM_2.5_ is the primary cause of visibility reduction in China and parts of the United States. When settling on the ground or water, these particles can exert different impacts on ecosystems depending on their chemical composition, including affecting ecological diversity, depleting soil nutrients and acidizing lakes and streams [8,9]. Inhalable PM_2.5_ can penetrate the respiratory system to aggravate respiratory symptoms [10,11] and increase mortality from cardiovascular and respiratory diseases after long-term exposure to PM_2.5_ [5,6,12]. In addition to the profound impacts on the environment and health, interactions between aerosols and radiation also affect climate change directly or indirectly in the long term [1,13–17]. To evaluate the long-term impacts of PM_2.5_ on the atmospheric environment, human health and climate change, it is critical to obtain historical PM_2.5_ datasets at high spatial-temporal resolutions. Nevertheless, the national hourly PM_2.5_ monitoring network from the Ministry of Ecology and Environment was not established until 2013. As a result of the limited PM_2.5_ observations, retrieving historical PM_2.5_ datasets is becoming a research hotspot.

With broad spatial coverage and relatively long observation periods (∼20 years), satellite-retrieved aerosol optical depth (AOD), a measure of the aerosol extinction of the solar beam, has been increasingly used to estimate large-scale PM_2.5_ concentrations for the past two decades [18–24]. For example, Huang *et al.* [20] predicted monthly PM_2.5_ concentrations on the North China Plain (NCP) from Multi-Angle Implementation of Atmospheric Correction (MAIAC) AOD using random forest that improved monthly prediction R^2^ (coefficient of determination) to 0.74. Additionally, using the MAIAC AOD, Xiao *et al.* [25] constructed monthly PM_2.5_ datasets from 2000 to 2018 to evaluate their spatial changes. Wei *et al.* [22] estimated daily PM_2.5_ across China using the space-time random forest approach with daily prediction R^2^ at 0.55. However, satellite-based AOD has some inherent limitations that are difficult to overcome. For example, a large proportion of non-random missing AOD due to cloud cover significantly affects data availability and generates biases [18,26]. Previous studies estimated that the missing AOD from MODIS (Moderate Resolution Imaging Spectroradiometer) accounted for 70%–90% of the total retrieval [26,27]. Apart from missing data, the sampling frequency of satellite-based AOD is typically limited to a maximum of twice a day. This kind of frequency makes it impossible for hourly PM_2.5_ assessments and leads to the underrepresentation of the average daily AOD. For model accuracy, the predictive accuracy for samples outside the training period is significantly lower than the validation accuracy, indicating that these models’ predictive capability is relatively weak. For example, although the R^2^ of 10-fold cross-validation (CV) on a daily scale can exceed 0.85, the R^2^ of the prediction is no more than 0.58 [21,22].

**Figure 1.**
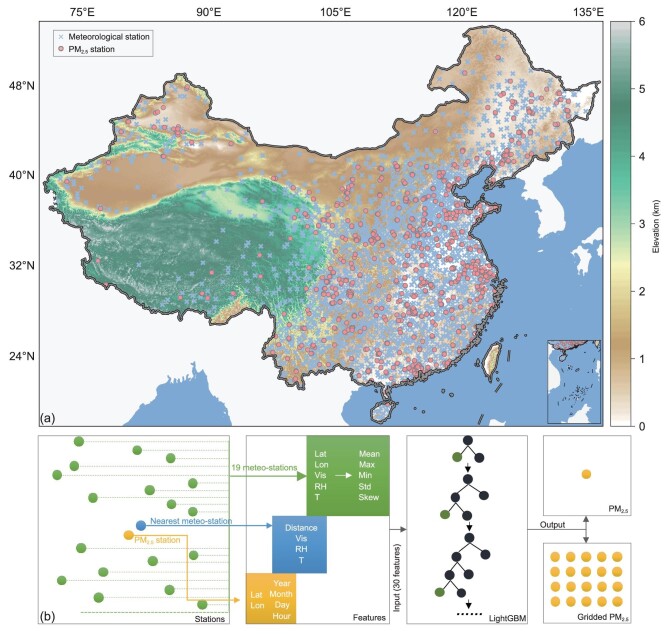
(a) Spatial distribution of 1440 PM_2.5_ stations and 2450 national meteorological stations across China and (b) a conceptual scheme for the extraction of spatial features and the development of our LightGBM model. Review drawing number: GS(2020)6868.

Compared with satellite-based AOD, horizontal visibility and other variables from surface meteorological observations have unique advantages in retrieving historical PM_2.5_. Surface meteorological observations that can be traced back to the 1950s have much more extended observation periods than satellite-based AOD observations. Surface observations are not disturbed by cloud cover and can continuously record hourly meteorological variables, which overcome the shortcomings of satellite-based AOD data that have massive missing values and low sample frequency. Unlike PM_2.5_ stations that are mainly located in cities, meteorological stations are distributed more evenly and densely. There are 2450 national meteorological stations and over 60 000 regional stations in China. This considerable magnitude has great potential to retrieve historical PM_2.5_ datasets at high spatial-temporal resolutions using visibility-dominated surface meteorological variables. For example, using daily meteorological observations, Gui *et al.* [28] have constructed a virtual ground-based PM_2.5_ network with XGBoost and achieved a better predictive capability with R^2^ values of 0.60 and 0.80 on daily and monthly scales, respectively. This work shows that visibility and other meteorological variables are promising for filling gaps in AOD-based PM_2.5_ [28]. Therefore, surface meteorological observations will continue to be used for retrieving PM_2.5_ but on an hourly scale in this study. We will employ a novel feature engineering approach to incorporate spatial effects and build a robust model of which the prediction capacity will improve significantly. The state-of-the-art machine-learning algorithm, LightGBM, will be used in this study to train the model based on over 30 million samples from meteorological observations at 2450 national stations from 2016 to 2018 (Fig. 1). The model performance will be evaluated using 10-fold CV. After validation, we will assess the predictive capability of this model using more than 10 million meteorological samples in 2019. Additionally, we will attempt to construct a densely gridded PM_2.5_ network by taking advantage of extracted spatial features.

## RESULTS AND DISCUSSION

### Model evaluation from hourly to yearly scales

In contrast to many machine learning models that are black boxes [29], LightGBM models can explain their predictions in a way that humans can understand. The decision-making process of our model was visualized using a feature-importance plot and a digraph representation of a specified tree. Figure S1 shows the relative importance of all the features used to train the model. Visibility from the nearest meteorological station is the most important feature that accounts for ∼7% of the overall importance. Approximately 6% of the overall importance is composed of distance that also significantly affects the model from a spatial perspective. Temporal features and other spatial features are also incorporated into the model, with relative importance ranging from 2% to 5%. Compared with the models in previous studies [20,22,26,28], our model does not heavily rely on one feature, e.g. AOD or visibility, but is able to integrate the influence of different features, particularly the spatial features that fully represent dimensional heterogeneity. For example, without visibility from the nearest station, the R^2^ value of observed and predicted PM_2.5_ in 2019 only decreases from 0.75 to 0.72 (Fig. S2). In contrast, the R^2^ value decreases more significantly to 0.65 when spatial features of visibility from surrounding stations are excluded (Fig. S2). To further gain an understanding of the decision process, we retrace the process of partitioning the notes on the training dataset by visualizing 1 of 1000 trees randomly in our model (Fig. S3). The tree node was split into child nodes based on the spatial visibility and then further divided based on the spatial relative humidity (RH) or visibility of the nearest station. It is clearly demonstrated how meteorological features, temporal features and spatial features play a role in our model.

**Figure 2.**
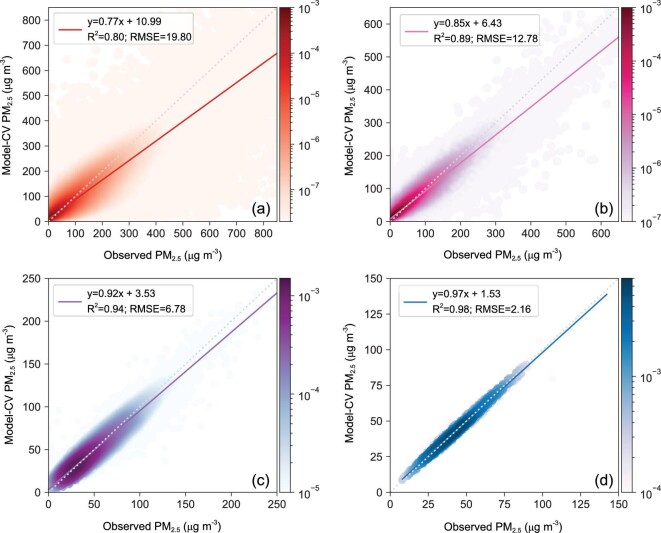
Density scatterplots of 10-fold CV results for (a) hourly (N = 31 863 778), (b) daily (N = 1 454 688), (c) monthly (N = 49 886) and (d) yearly (N = 1440) PM_2.5_ from 2016 to 2018 across China (colors show probability distribution densities).

Our model's performance was evaluated with 10-fold CV using 31 863 778 hour-by-hour training data across China from 2016 to 2018. As shown

in Fig. 2, the overall R^2^ and root-mean-square error (RMSE) for hourly PM_2.5_ estimations are 0.80 and 19.80 μg m^−3^, respectively, which are in close agreement with the fitting results with the R^2^ value of 0.80 and the RMSE value of 19.60 μg m^−3^ (Fig. S4). This finding indicates that this model can effectively avoid overfitting and achieve high and stable accuracy in estimating hourly PM_2.5_ concentrations. The performance of our model is even better for PM_2.5_ estimations on larger timescales. For daily estimations with 1 454 688 samples, the overall  R^2^ and RMSE are 0.89 and 12.78 μg m^−3^, respectively. For monthly

and yearly estimations, the overall  R^2^ values increase to 0.94 and 0.98, respectively, and the RMSE values decrease to 6.78 μg m^−3^ and 2.16 μg m^−3^, respectively. To better present our model's performance, we compared our CV scores with those in recent studies that predicted PM_2.5_ across China. As shown in Table 1, our model outperformed all of the other models in the R^2^ and RMSE for model validation from daily to yearly scales and allowed for unprecedented hour-by-hour evaluation with R^2^ (0.80) even better than most of the other R^2^ values on a daily scale (0.41∼0.85) [20–22,25,28,30–38].

**Table 1.** Statistics for the comparison of the validation performance and predictive capability of different models from hourly to yearly scales.

Basic information			Model validation								Predictive capability							
			Hourly		Daily		Monthly		Yearly		Hourly		Daily		Monthly		Yearly	
Primary predictor	Model	References	R^2^	RMSE	R^2^	RMSE	R^2^	RMSE	R^2^	RMSE	R^2^	RMSE	R^2^	RMSE	R^2^	RMSE	R^2^	RMSE

AOD	GWR	[30]	-^a^	-	0.64	32.98	-	-	-	-	-	-	-	-	-	-	-	-
AOD	Stage-1	[32]	-	-	0.78	27.99	-	-	-	-	-	-	-	-	-	-	-	-
AOD	Stage-2		-	-	0.79	27.42	-	-	-	-	-	-	0.41	-	0.73	-	0.79	-
AOD	TSAM	[31]	-	-	-	-	-	-	0.80	22.75	-	-	-	-	-	-	-	-
AOD	GWR	[33]	-	-	0.79	18.60	-	-	-	-	-	-	-	-	-	-	-	-
Visibility	ER	[38]	-	-	0.42	-	-	-	-	-	-	-	0.38	-	-	-	-	-
AOD	Gaussian	[36]	-	-	0.81	21.87	-	-	-	-	-	-	-	-	-	-	-	-
AOD	GRNN	[34]	-	-	0.67	20.93	-	-	-	-	-	-	-	-	-	-	-	-
Visibility	LMEM	[35]	-	-	-	-	0.71	25.62	-	-	-	-	0.60	-	0.71	-	-	-
AOD	GTWR	[37]	-	-	0.80	18.00	-	-	-	-	-	-	0.47	12.03	-	-	-	-
Merra-2 PM_2.5_	RF	[20]	-	-	-	-	0.88	14.89	-	-	-	-	-	-	0.74	17.80	0.76	11.35
AOD	Ensemble	[21]	-	-	-	-	0.79	21.00	-	-	-	-	0.58	29.00	0.76	15.70	-	-
AOD	MLR	[22]	-	-	0.41	20.04	-	-	-	-	-	-	0.38	21.97	-	-	-	-
	GWR		-	-	0.53	23.28	-	-	-	-	-	-	0.44	26.47	-	-	-	-
	Stage-1		-	-	0.65	19.50	-	-	-	-	-	-	0.31	27.73	-	-	-	-
	Stage-2		-	-	0.71	8.59	-	-	-	-	-	-	0.35	27.65	-	-	-	-
	RF		-	-	0.81	17.91	-	-	-	-	-	-	0.53	28.09	-	-	-	-
	STRF		-	-	0.85	15.57	-	-	-	-	-	-	0.55	27.38	-	-	-	-
AOD	Ensemble	[25]	-	-	-	-	0.91	9.30	-	-	-	-	-	-	0.78	14.00	-	-
Visibility	Xgboost	[28]	-	-	0.79	15.75	0.92	6.75	-	-	-	-	0.60	25.34	0.80	14.75	0.83	10.10
Visibility	LightGBM	This study	0.80	19.80	0.89	12.78	0.94	6.78	0.98	2.16	0.75	19.19	0.84	13.82	0.88	8.39	0.87	5.55

^a^‘-’ indicates no data.

### Robust prediction of hourly PM_2.5_ across China

**Figure 3.**
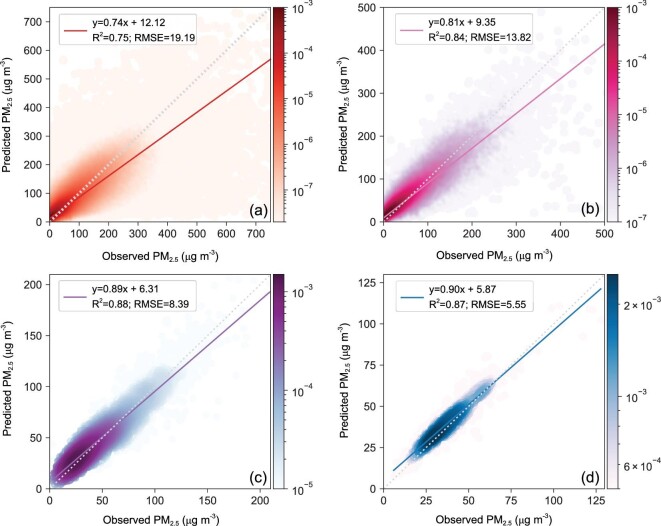
Density scatterplots of observed PM_2.5_ and predicted PM_2.5_ on (a) hourly (N = 10 522 939), (b) daily (N = 477 867), (c) monthly (N = 49 886) and (d) yearly (N = 1440) timescales in 2019 across China (colors are probability distribution densities).

Our model's predictive capability, which is crucial for retrieving historical PM_2.5_ datasets, was evaluated using 10 522 939 ‘unseen’ samples from 2019. Figure 3 presents the overall correlations of model-predicted PM_2.5_ and observed PM_2.5_ on different timescales. For hourly PM_2.5_ prediction, the overall R^2^ and RMSE are 0.75 and 19.19 μg m^−3^, respectively, which are closely consistent with the 10-fold CV results (Fig. 2a). This excellent relevance indicates that our model has a robust predictive capability that can construct hourly historical PM_2.5_ datasets feasibly and accurately. The predictive power of this model is even better for PM_2.5_ prediction on larger timescales. For daily estimations with 477 867 samples, the overall R^2^ and RMSE are 0.84 and 13.82 μg m^−3^, respectively. For monthly and yearly estimations, the overall  R^2^ values increase to 0.88 and 0.87, respectively, and the RMSE values decrease to 8.39 μg m^−3^ and 5.55 μg m^−3^, respectively. To better evaluate the predictive power of our model, we compared our predictive scores with those in recent studies (Table 1). As mentioned above, the R^2^ and RMSE of the predictions are significantly worse than those of the 10-fold CV, particularly for the models based on satellite-retrieved AOD. The best predictive R^2^ of AOD-based models is only 0.58 on a daily scale, which indicates that there will be potential biases that cannot be ignored when we estimate PM_2.5_ datasets using those models. Compared with the other models in Table 1, our model can provide unprecedented hour-by-hour predictions on PM_2.5_ and gains considerable advantages in predictive capacity from daily to yearly scales. These advantages mainly result from the incorporation of spatial features from 19 surrounding meteorological stations. If these spatial features are removed, the predictive capacity of our model is significantly reduced, with the R^2^ values decreasing to 0.61 and 0.72 on hourly and daily scales, respectively. This kind of performance is only slightly better than that of models in previous studies.

**Figure 4.**
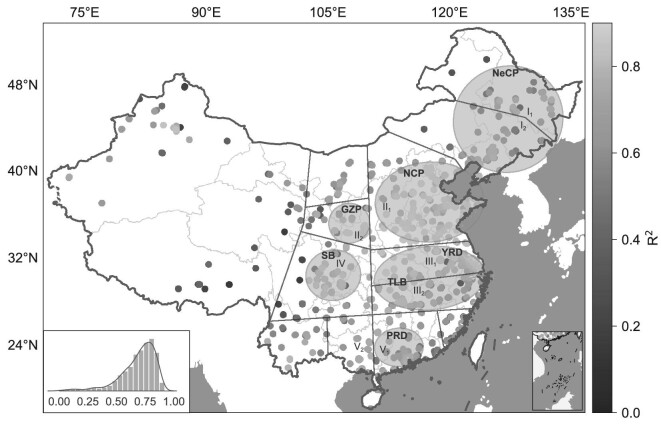
Spatial distribution of R^2^ between observed PM_2.5_ and predicted PM_2.5_ on an hourly scale in 2019 across China. Eastern China is divided into similar visibility-changing regions with black lines as defined in Zhang *et al.* [39], and key polluted regions marked with shaded circles. Review drawing number: GS(2020)6868.

To evaluate the predictive capacity of our model in different regions of China, we obtained the spatial distribution of R^2^ values between observed PM_2.5_ and model-predicted PM_2.5_ on an hourly scale (Fig. 4). Five key polluted regions were selected as focuses based on long-term trends in visibility [39], including (i) the NCP and the Guanzhong Plain (GZP) in northern China; (ii) the Yangtze River Delta (YRD) region and the Two Lakes Basin (TLB) along the middle and lower reaches of the Yangtze River; (iii) the Pearl River Delta (PRD) region in southern China; (iv) the Sichuan Basin (SB) in southwestern China; and (v) the Northeast China Plain (NeCP) [39,40]. As shown in Fig. 4, the predictive capacity of our model is remarkable in the five regions and is much better in more polluted regions. Among these regions, the model presents the most impressive predictive performance on the NCP, with R^2^ values generally more than 0.80. Following the NCP, the model also shows high accuracy in PM_2.5_ prediction on the GZP, with R^2^ values over 0.80. The SB, which is a cloudy basin with more than 70% of AOD values missing, still presents reliable predictive performance, with R^2^ of ∼0.75. On the YRD and TLB, the R^2^ values fluctuate between 0.65 and 0.90 but still exceed 0.80 in most cases. On the NeCP and PRD, the predictive performance is also acceptable with R^2^ values of approximately or over 0.70. The regional differences of R^2^ in these five regions might be affected by three factors, including pollution levels, RH and the distribution of meteorological stations. Under low levels of pollution, PM_2.5_ concentrations are not closely related to visibility that is the most important feature for our model. As pollution levels increase, visibility is increasingly affected by PM_2.5,_ and the non-linear relationship between these two variables might be more easily built by our model. As a result, the NCP and the GZP, which experience the most severe aerosol pollution, exhibit the best performance of predictive power among all the five regions. In addition to PM_2.5_, visibility is also affected by RH that can enhance aerosol hygroscopic growth [41]. RH exhibited significant differences between northern and southern China. In northern China, RH remains relatively low, and haze frequently occurs; while in southern China, RH remains relatively high, and mist or fog often occurs [42]. The non-linear relationship between visibility and PM_2.5_ is more complicated in southern China and might be more difficult to build by our model. As a result, in southern China, including the PRD, the TLB, the YRD and the SB, the R^2^ is slightly lower than that in northern China, including the NCP and the GZP. It is worth noting that the R^2^ is also slightly lower in parts of northern China, including Inner Mongolia and the NeCP, which might result from the sparsity of meteorological stations that cannot truly reflect real situations. In contrast to the model performance in these five regions, at dozens of stations on the Tibet Plateau and its surrounding areas the model shows poor performance in hourly PM_2.5_ prediction, with R^2^ less than 0.40. The 25 stations with the lowest R^2^ values were extracted to explore the causes of the low R^2^ values, while another 25 stations with the highest R^2^ values were used as contrasts. We found that the poor predictive capability at these stations mainly results from extremely low PM_2.5_ concentrations (<20 μg m^−3^) that cannot effectively be reflected by visibility and long-distance surrounding meteorological stations (Fig. S5). The nearest meteorological station is ∼80 km on average away from the PM_2.5_ station, and the 20th nearest meteorological station is 300 km away (Fig. S5). Such a distance indicates that surrounding meteorological stations cannot truly reflect real situations around PM_2.5_ stations. Nevertheless, this disadvantage will be overcome when we incorporate regional meteorological stations in the future.

Previous studies also revealed that training regional models for each region can improve model performance due to significant spatial

heterogeneity in relationships between PM_2.5_ and meteorological variables [21]. Therefore, we selected three representative regions and trained regional models for each region, respectively. Figure S6 shows performance differences between the national and regional models on the NCP, the YRD and the Tibet Plateau. Compared with regional models, the national model performed almost equally well on the NCP, slightly better on the YRD, and slightly worse on the Tibet Plateau (Fig. S6). This finding indicates that regional models for each region cannot provide more accurate results. The spatial heterogeneity might have been incorporated into our national model by taking advantage of spatial features.

Given our model's hourly predictions, we assessed its predictive capacity for diurnal variations in PM_2.5_. Figure S7 shows a clear diurnal variation in observed PM_2.5_ across China. PM_2.5_ concentrations significantly increased after 20:00 (Beijing Time, BJT) and decreased after 12:00. This diurnal variation is well captured by our PM_2.5_ predictions, which are almost the same as the PM_2.5_ observations (Fig. S7). Due to the diurnal PM_2.5_ variations, using satellite-based AOD as the daily average, which is obtained twice a day (using MODIS as an example), will inevitably overestimate the actual daily conditions.

The robust prediction of our model can also be demonstrated by the hourly time series of observed and predicted PM_2.5_ at several representative stations in Beijing, Shijiazhuang, Xi’an, Chengdu and Shanghai. As shown in Fig. S8, this model accurately predicts low values, high values and variation trends in both low-pollution and high-pollution areas.

### Gridded PM_2.5_ networks at high spatial-temporal resolutions

**Figure 5.**
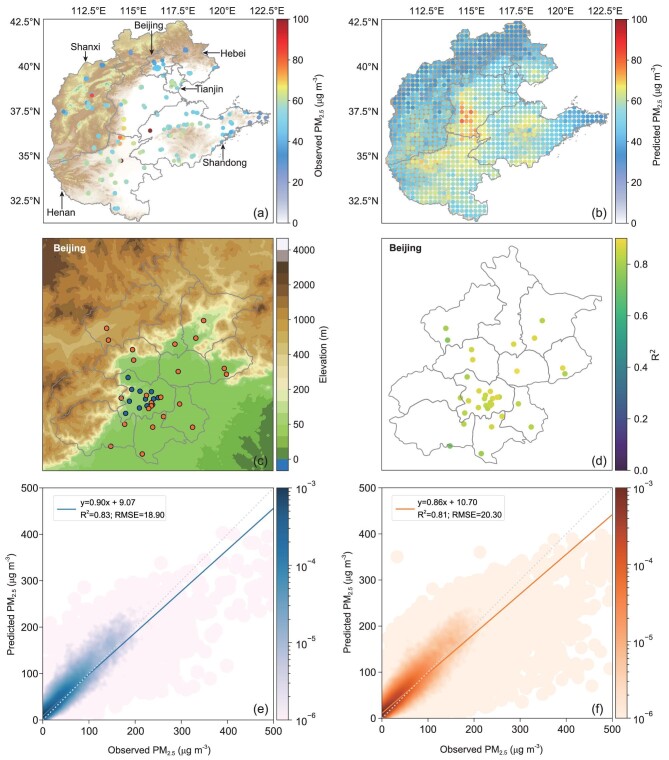
Spatial distribution of (a) observed PM_2.5_ and (b) predicted gridded networks of PM_2.5_ on a yearly scale on the North China Plain; (c) the distributions of 12 national stations (blue) that have been used during the training process and 23 regional stations (orange) that are untouched during the training process in Beijing; (d) the distribution of R^2^ for both national and regional stations; (e) density scatterplots of observed PM_2.5_ and predicted PM_2.5_ for 12 national stations on an hourly scale and (f) density scatterplots of observed PM_2.5_ and predicted PM_2.5_ for 23 untouched regional stations on an hourly scale. Review drawing number: GS(2020)6868.

The distribution of PM_2.5_ stations is uneven, with most stations located in urban areas in eastern China. In contrast, meteorological stations are distributed more evenly across China at a higher density. By taking advantage of spatial features from surrounding meteorological stations, our model can construct densely-gridded PM_2.5_ networks at high temporal resolutions. For better visualization, we set the grid point precision to 0.25°, which can be further increased if required with consideration for numbers, distribution density and spacing distance of meteorological stations in the target area. Figure 5a and b shows the distribution of observed PM_2.5_ stations and gridded PM_2.5_ networks from our prediction. The gridded networks accurately correspond to observed PM_2.5_ concentrations and provide more detailed information on spatial distributions. We found that low PM_2.5_ concentrations occur in the northwestern mountain areas and that several pollution centers existed in the west and south, as well as in the hinterlands of the mountains. The region in southern Hebei and north-central Henan experience the widest and highest pollution among all the polluted centers. Compared with that region, the region in central Shandong undergoes less severe pollution. Another two centers that experience weak pollution at small ranges are located in eastern Hebei and the hinterlands in Shanxi. Apart from the assessment on a yearly scale, gridded networks from diurnal variations are illustrated in Fig. S9. Compared with observations in Fig. S10, the gridded networks present the diurnal variations in a better and clearer way.

The accuracy of the gridded PM_2.5_ networks depends on whether the model can well predict PM_2.5_ concentrations at locations outside the scope of the training stations. In Beijing, 23 regional PM_2.5_ stations were untouched during the training process (Fig. 5c) and thus can be used to evaluate this kind of accuracy. Hourly PM_2.5_ concentrations in these stations were predicted by the model and compared with PM_2.5_ observations in 2019. As shown in Fig. 5d, the R^2^ values exceed 0.75 at 22 of 23 regional stations and do not exhibit significant differences between national and regional stations.

 

For hourly PM_2.5_ concentrations at all 23 regional stations, the R^2^ and RMSE are 0.81 and 20.30, respectively, which are just slightly weaker than those (R^2 ^= 0.83, RMSE = 18.90) at 12 national stations (Fig. 5e and f). These results indicate that our model is able to well predict PM_2.5_ concentrations at locations both inside and outside the scope of the training stations.

Several polluted stations that almost coincide with other stations are not incorporated into our gridded networks. This phenomenon is mainly due to our grid precision settings and will be effectively resolved if sufficiently high precision is set. Furthermore, it might be difficult for us to construct gridded networks in western China where meteorological stations are scarce, but this will be significantly improved when we introduce regional meteorological stations in the future. The significant increase in the number of meteorological stations will enable us to build densely-gridded networks on an hourly scale.

## CONCLUSION

For retrieving historical PM_2.5_ datasets, satellite-based AOD has some inherent limitations that are difficult to overcome, i.e. massive missing values due to cloud cover, low sampling frequency and weak predictive capability for data outside the training period. Here, hourly meteorological observations with over 40 000 000 samples were employed to overcome the disadvantages of satellite-based retrieval. Developing a novel feature engineering approach to extract spatial features of surrounding stations, we built a LightGBM model that outperformed previous models regardless of validation performance or predictive capability. The R^2^ and RMSE of the 10-fold CV of our model are 0.80 and 19.80 μg m^−3^ on an hourly scale and 0.89 and 12.78 μg m^−3^ on a daily scale, respectively. This model can even achieve unprecedented hour-by-hour PM_2.5_ predictions with high and stable accuracy. For hourly PM_2.5_ prediction, the overall R^2^ and RMSE are 0.75 and 19.19 μg m^−3^, respectively. For daily, monthly and yearly PM_2.5_ predictions, the R^2^ values are 0.84, 0.88 and 0.87, respectively, and the RMSE values are 13.82 μg m^−3^, 8.39 μg m^−3^ and 5.55 μg m^−3^, respectively. By taking advantage of spatial features, our model can also construct hourly gridded networks of PM_2.5_ at high spatial resolutions that provide more detailed information on spatial distribution. Our results show that this model has great potential in reconstructing historical PM_2.5_ datasets at high spatial-temporal resolutions and retrieving real-time gridded PM_2.5_ networks across China. However, this model still has some weaknesses, with the main weakness being the poor performance in predicting hourly PM_2.5_ in dozens of stations in western China where meteorological stations are sparse. This disadvantage will be effectively overcome when regional meteorological stations are incorporated. In the future, we will employ this model to hindcast two sets of historical PM_2.5_ datasets from the 1950s. One dataset will be for existing PM_2.5_ stations and the other for gridded networks of PM_2.5_. Then, we will incorporate regional meteorological stations to improve our model's precision and then use the model to retrieve gridded networks of PM_2.5_ at high spatial-temporal resolutions. This will serve to overcome the disadvantages of existing PM_2.5_ stations that are unevenly distributed and far fewer than meteorological stations in number. In addition, the retrieved historical PM_2.5_ datasets will be assimilated into models to produce long-term re-analysis that incorporates interactions between aerosols and the physical processes of the climate system. This re-analysis will facilitate investigating aerosols’ impacts on society, epidemiology and climate change.

## MATERIALS AND METHODS

### Observational data

This study used ground-based PM_2.5_ observations at ∼1600 national stations across China from 2016 to 2019 (Fig. 1a). The hourly PM_2.5_ data were archived at the China National Environmental Monitoring Center (CNEMC, http://www.cnemc.cn, 11 October 2020). We conducted a series of quality controls to produce high-quality data. A total of 1440 stations met the quality criterion of having at least 60% of valid data and were retained for this study. Severe outliers that were abnormally higher than surrounding data were effectively removed using a method that compared the differences between hourly PM_2.5_ and a five-point moving average. After several tests, the threshold of 150 μg m^−3^ was effective in eliminating outliers (Fig. S11). With this threshold, ∼3% of the hourly PM_2.5_ was removed. A total of 42 386 717 samples remained for model development and application. There are also 23 regional PM_2.5_ stations in Beijing in addition to 12 national stations (https://quotsoft.net, 11 October 2020). Hourly PM_2.5_ data from these 23 stations were not used for model training but for evaluating predictive capability.

National ground-based surface meteorological observations from 2016 to 2019 were archived at the National Meteorological Information Center of the China Meteorological Administration. Similarly, we only use the stations with valid values over 60%. There were 2450 stations remaining in total (Fig. 1). Three meteorological variables, including visibility, RH and temperature, were selected from the surface observations as the main predictors to develop the LightGBM model.

### Feature engineering approach

The longitude, latitude and time variables (year, month, day and hour) of PM_2.5_ stations were selected as features at first (Fig. 1b). Then we matched each PM_2.5_ station with its nearest meteorological station and added the visibility, RH, and temperature from that meteorological station as features (Fig. 1b). The distance between these two stations was also added as a feature. Previous studies have shown that the pollution levels at one station are largely affected by the surrounding environment, i.e. pollution transport is the primary cause of early pollution formation in Beijing [43,44]. Therefore, spatial effects need to be considered for better accuracy. Here, we developed a novel feature engineering approach that extracts spatial features to incorporate the surrounding environment's effects. Specifically, the nearest 20 meteorological stations for each PM_2.5_ station were matched, and the nearest station that had already been used was excluded. We extracted five variables from the remaining 19 meteorological stations, including latitude, longitude, visibility, temperature and RH. For each variable, we calculated the mean values, maximum values, minimum values, standard deviation and skewness values in order and added them as features. After all the features were obtained, feature selection was performed to reduce training time and improve accuracy. We employed a relatively small sample dataset (1/3 of training data) to train the model and obtain each feature's importance in the dataset. According to the highest to lowest importance, we selected the top 30 features for the following model fitting, validation and evaluation. These features included visibility, distance and other spatial features.

### The LightGBM model and its development

LightGBM is a state-of-the-art gradient-boosting framework that uses tree-based learning algorithms [45]. It is designed with faster training speed, lower memory usage, better accuracy and capability of handling large-scale data [45]. Until now, it could achieve slightly higher accuracy with much faster speed compared to XGBoost. Therefore, it was more appropriate to use LightGBM in our study, which included more than 40 million samples. Two metrics, R^2^ and RMSE, were employed to quantify the quality of predictions. Then we trained the LightGBM model with 30 features and PM_2.5_ labels from 2016 to 2018. To achieve a better performance, we performed hyperparameter tuning with a randomized search CV (RSCV) optimized by a randomly cross-validated search on parameter settings. In contrast to grid search CV (GRCV) that tries out all parameter values, RSCV only uses a fixed number of parameter settings to obtain a local optimal solution. This solution, which saves much computation time, was more realistic for our study. Based on RSCV and our tuning experiences, we finally selected the following hyperparameters: max_depth = 16, num_leaves = 127, min_data_in_leaf = 10, learning_rat = 0.05, feature_fraction = 0.80, bagging_fraction = 0.80, bagging_freq = 5, max_bin = 255, lambda_l1 = 0.5, lambda_l2 = 0.5 and num_boost_round = 1000. To evaluate the model performance, we performed 10-fold CV on the training data. Thirty features and PM_2.5_ labels from 2016 to 2018 were randomly divided into 10 sets. For each of the 10 folds, a model was developed using the other nine folds as training data, and subsequently, the resulting model was validated with the data of this fold. The R^2^ and RMSE reported by 10-fold CV reflect the averages of the values calculated in the loop. After the model was built, 30 meteorological features in 2019 were input into this model to generate PM_2.5_ predictions for further evaluating our model's predictive capability on hourly, daily, monthly and annual scales. The flow of building the LightGBM model is illustrated as a conceptual figure (Fig. 1b).

### Construction of gridded networks

The gridded meteorological input was generated to construct gridded PM_2.5_ networks. The detailed process was demonstrated using the NCP as an example. First, we define an area with latitude from 30°N to 45°N and longitude from 110°E to 125°E, which can cover the whole NCP. This area was then gridded at 0.25° intervals, and 3600 grid points were generated with latitude and longitude as features. The nearest meteorological station was matched for each point, and four variables from this station are added to the point as features, including visibility, RH, temperature and distance. Spatial variables from the surrounding 19 stations are also added to the point as features. After that, we generate 3600 grid points with geological information, meteorological variables and spatial features. As these grid points are input into our model, gridded PM_2.5_ networks are constructed.

Since 23 regional PM_2.5_ stations in Beijing are excluded during the training process, hourly PM_2.5_ concentrations in 2019 at these stations can be used to evaluate our model's performance. For each station, four variables of its nearest meteorological station and spatial variables from surrounding stations in 2019 are added to the station as input features. The generated input datasets at 23 regional stations are input into our model to produce predicted PM_2.5_ that were further compared with observed PM_2.5_.

## DATA AVAILABILITY

The data that support the findings of this study are available from the corresponding authors upon reasonable request.

## Supplementary Material

nwaa307_Supplement_File
