# Supplementary material for: Robust prediction of hourly PM2.5 from meteorological data using LightGBM
Source: Natl Sci Rev. 2021 Jan 5;8(10):nwaa307. doi: 10.1093/nsr/nwaa307 (PMC8566180; doi:10.1093/nsr/nwaa307)
Supplement: nwaa307_Supplement_File [file nwaa307_supplement_file.pdf]

Supplementary Materials for

Robust prediction of hourly $PM_{2.5}$ from meteorological data using LightGBM

Junting Zhong[1, 2], Xiaoye Zhang[1, 3*], Ke Gui[1*], Yaqiang Wang[1], Huizheng Che[1], Xiaojing Shen[1], Lei Zhang[1], Yangmei Zhang[1], Junying Sun[1], Wenjie Zhang[1]

[1]State Key Laboratory of Severe Weather & Key Laboratory of Atmospheric Chemistry of CMA, Chinese Academy of Meteorological Sciences, Beijing, 100081, China

[2]University of Chinese Academy of Sciences, Beijing 100049, China

[3]Center for Excellence in Regional Atmospheric Environment, IUE, Chinese Academy of Sciences, Xiamen, 361021, China.

Correspondence to: Xiaoye Zhang (xiaoye@cma.gov.cn) and Ke Gui (guik@cma.gov.cn)

This document includes Supplementary Figures S1-11

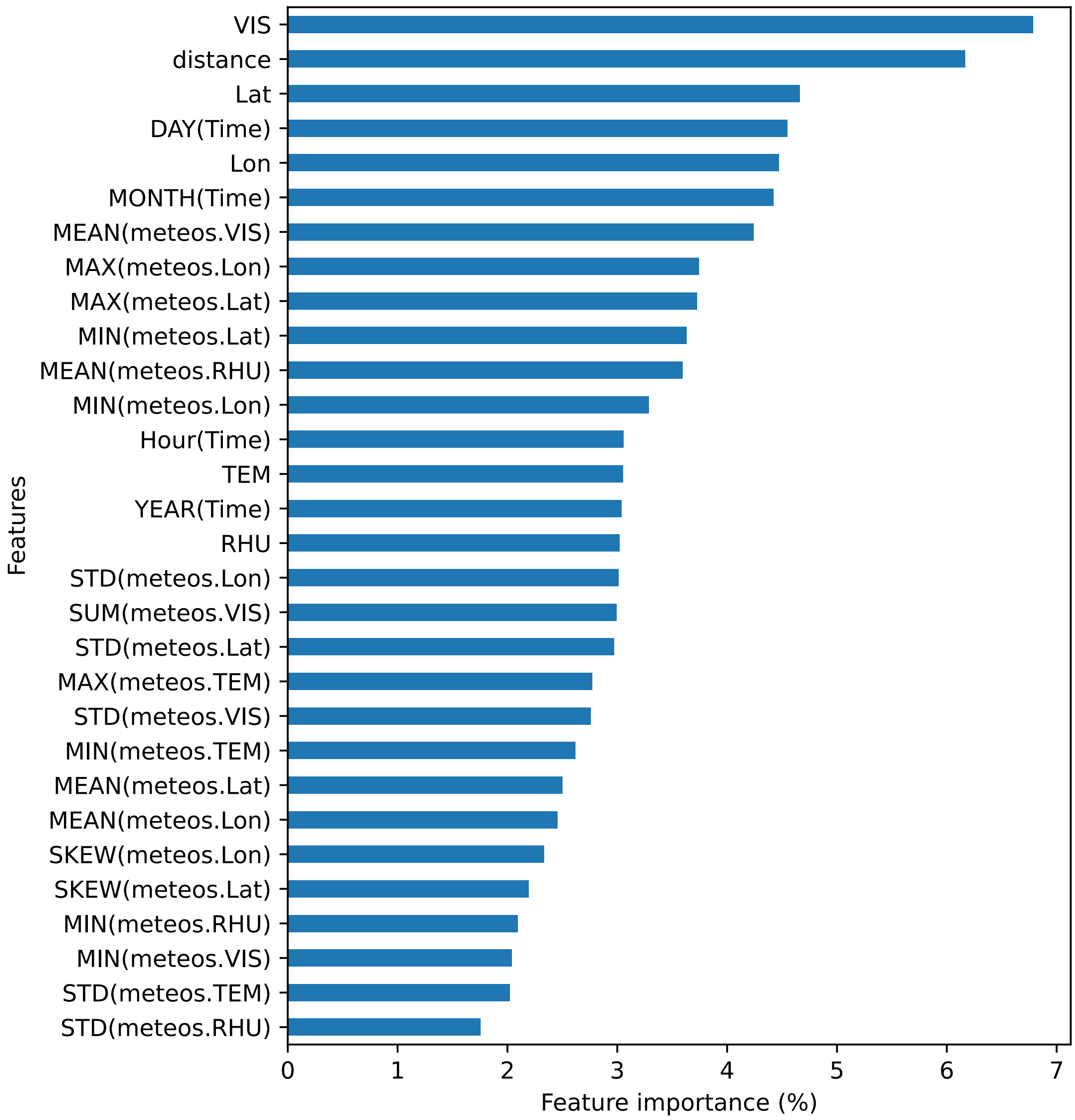

Fig. S1 Relative importance of all the features used in our LightGBM model

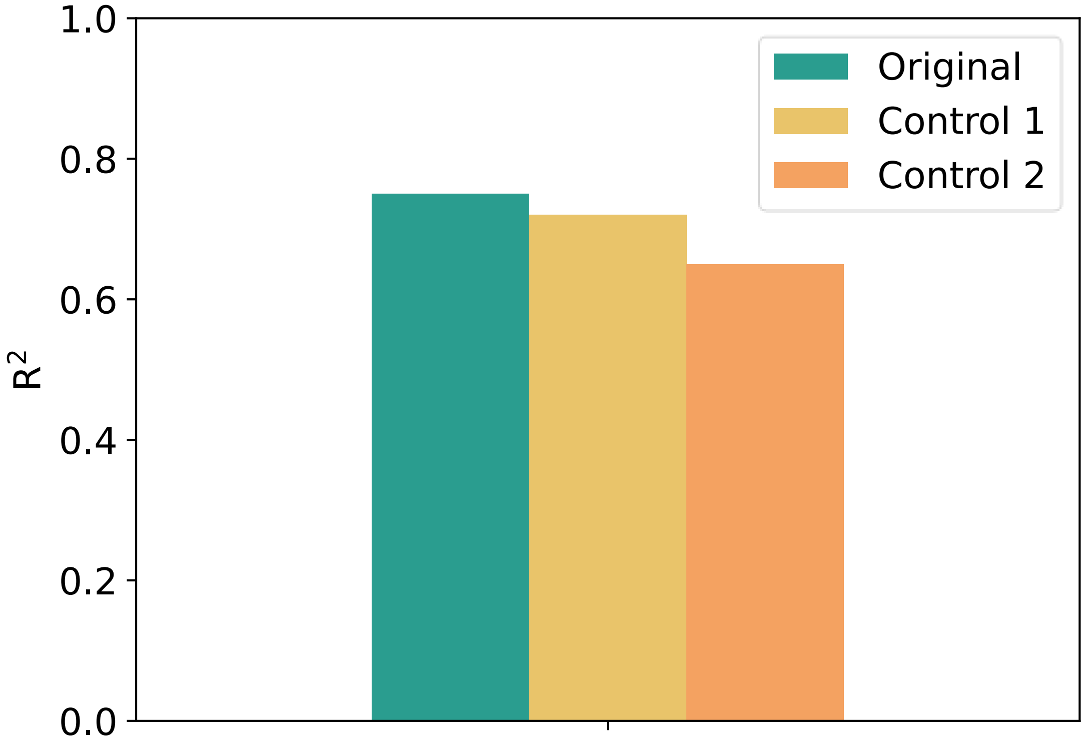

Fig. S2 The R^2 between observed $PM_{2.5}$ and predicted $PM_{2.5}$ from models trained with different features. The original group used all 30 features in Fig. S1; the first control group (Control 1) only removed visibility from the nearest station; and the second control group (Control 2) only removed spatial features of visibility from surrounding 19 stations.

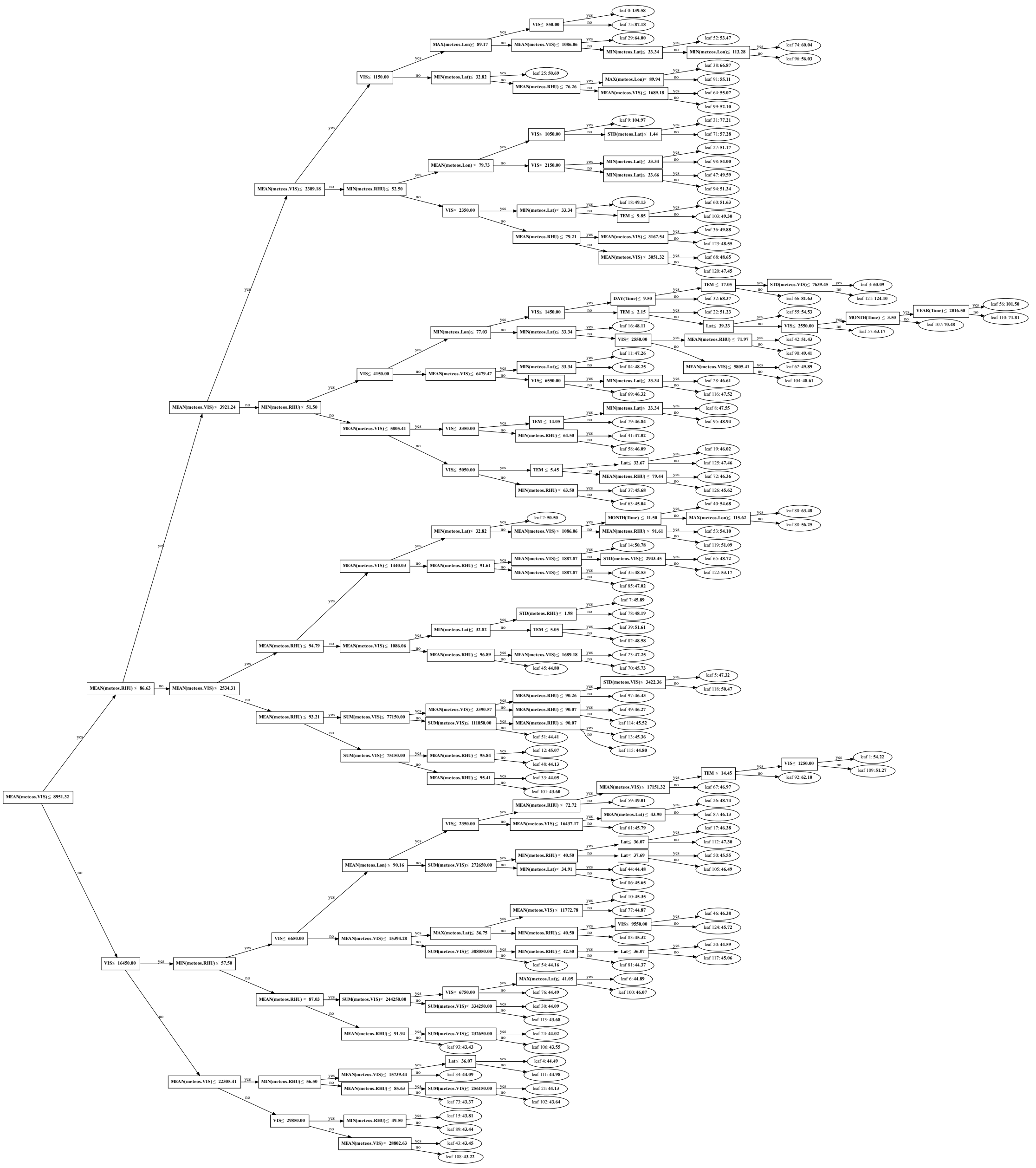

Fig. S3 A digraph representation of 1 of 1000 trees in our LightGBM model.

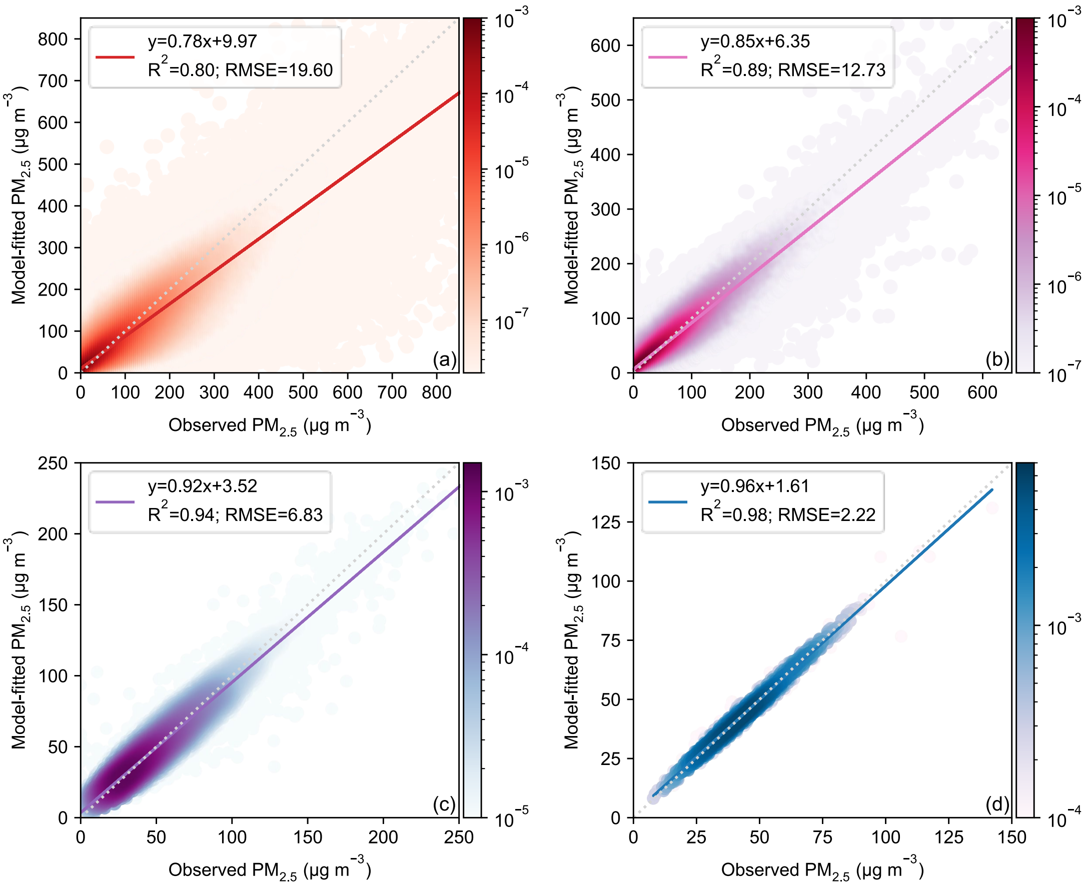

Fig. S4 Density scatterplots of fitting results for (a) hourly, (b) daily, (c) monthly, and (d) yearly $PM_{2.5}$ from 2016 to 2018 across China (colors show probability distribution densities).

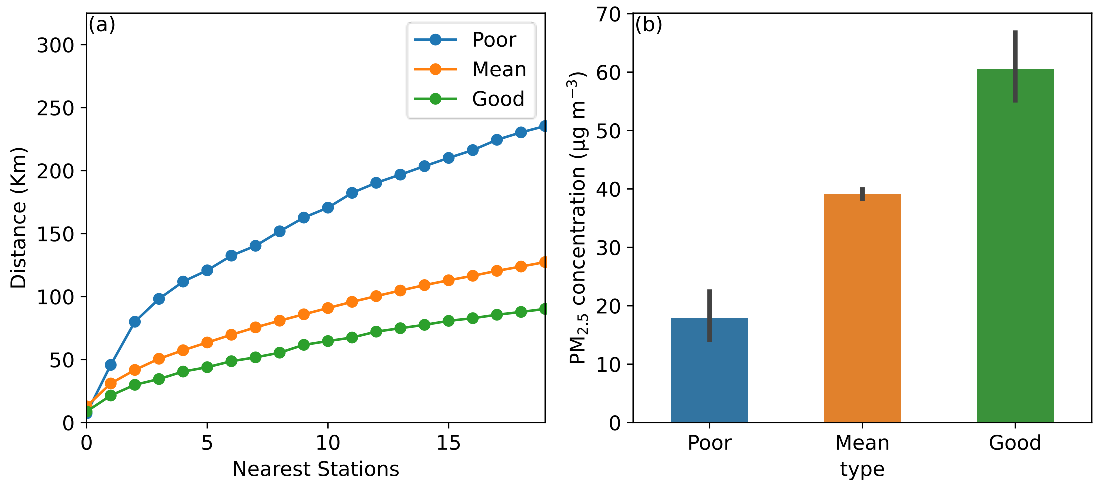

Fig. S5 (a) The mean distance between different $PM_{2.5}$ stations and 20 nearest meteorological stations, and (b) the mean $PM_{2.5}$ mass concentrations of different types ("poor": the 25 stations with lowest R^2 for prediction; "mean": all the $PM_{2.5}$ stations; and "good": 25 stations with highest R^2 used as a contrast).

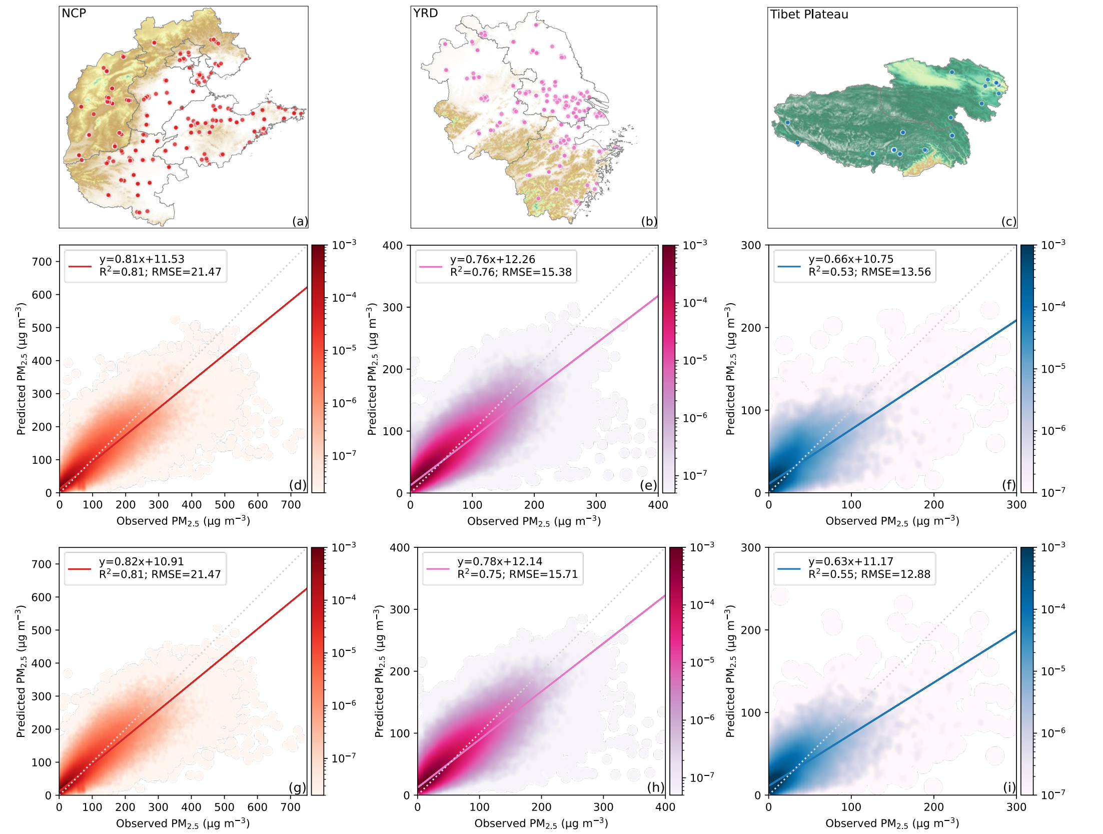

Fig. S6 (a, b, c) Spatial distribution of $PM_{2.5}$ stations on the North China Plain (NCP), the Yellow River Delta (YRD), and the Tibet Plateau (including Qinghai and Tibet); (d, e, f) density scatterplots of observed $PM_{2.5}$ and predicted $PM_{2.5}$ on an hourly scale in 2019 based on a unified model for these three regions, respectively; and (g, h, i) density scatterplots of observed $PM_{2.5}$ and predicted $PM_{2.5}$ on an hourly scale in 2019 for these three regions, respectively, based on models trained separately for each region.

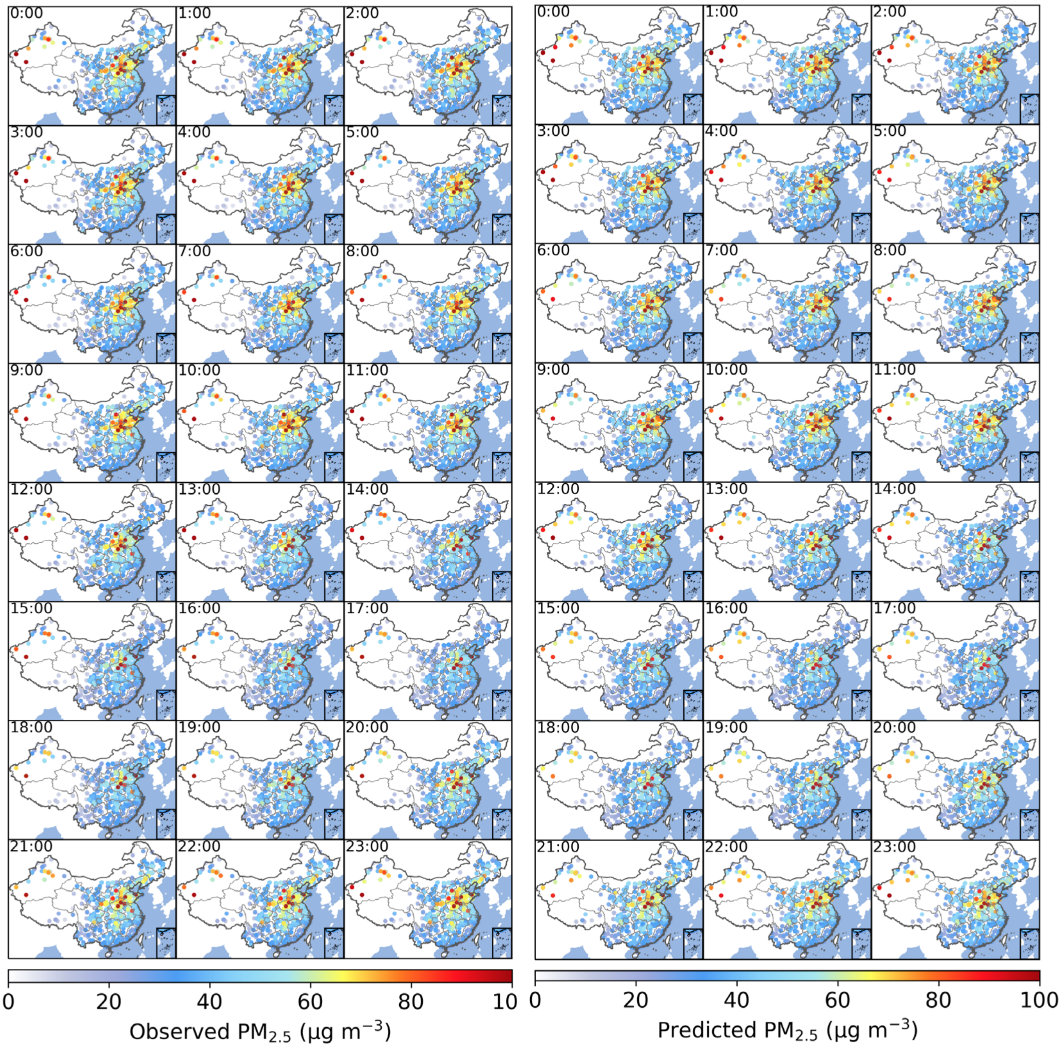

Fig. S7 Diurnal variations in the spatial distribution of observed $PM_{2.5}$ (left panel) and predicted $PM_{2.5}$ (right panel) in 2019 across China.

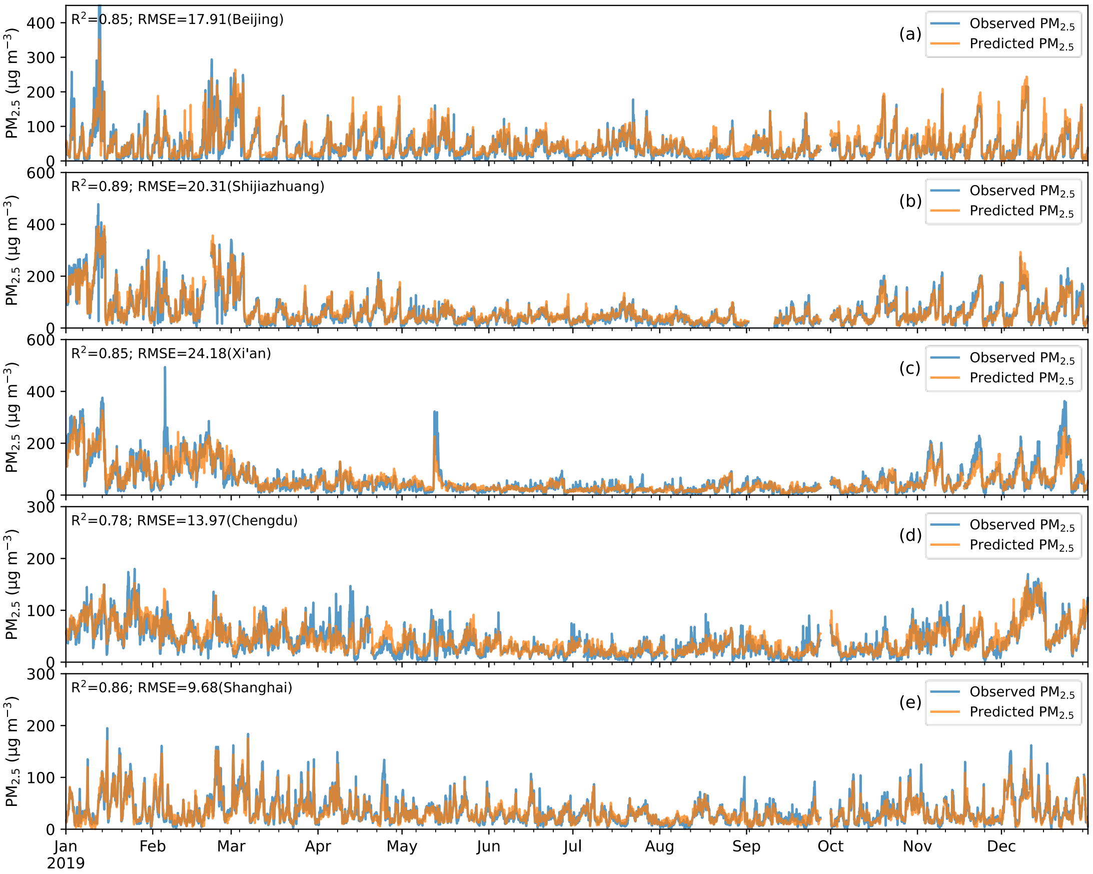

Fig. S8 Hourly time series of observed and predicted $PM_{2.5}$ in several representative stations in Beijing, Shijiazhuang, Xi'an, Chengdu, and Shanghai.

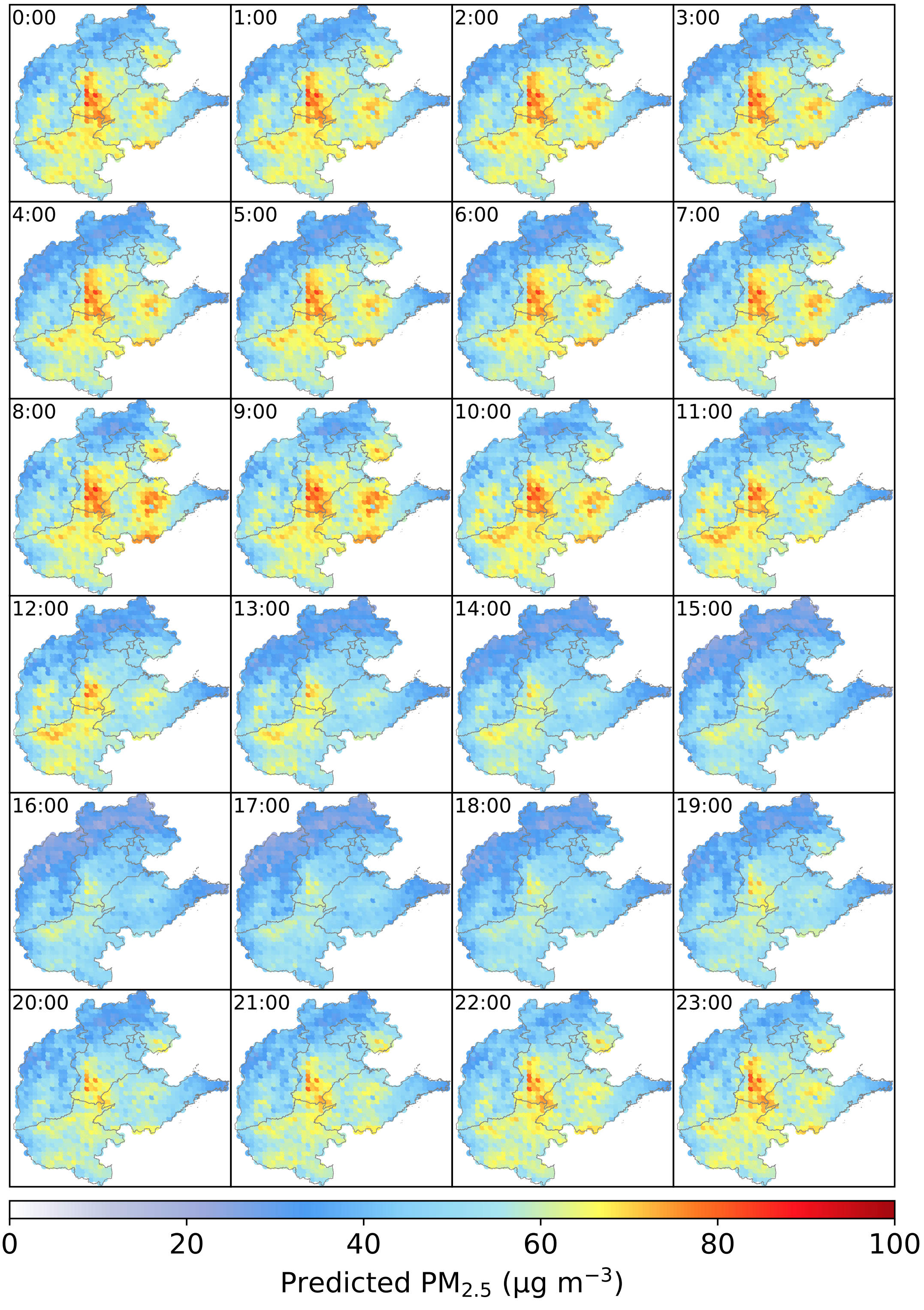

Fig. S9 Diurnal variations in the spatial distribution of the gridded networks of $PM_{2.5}$ from our prediction in 2019 across the North China Plain.

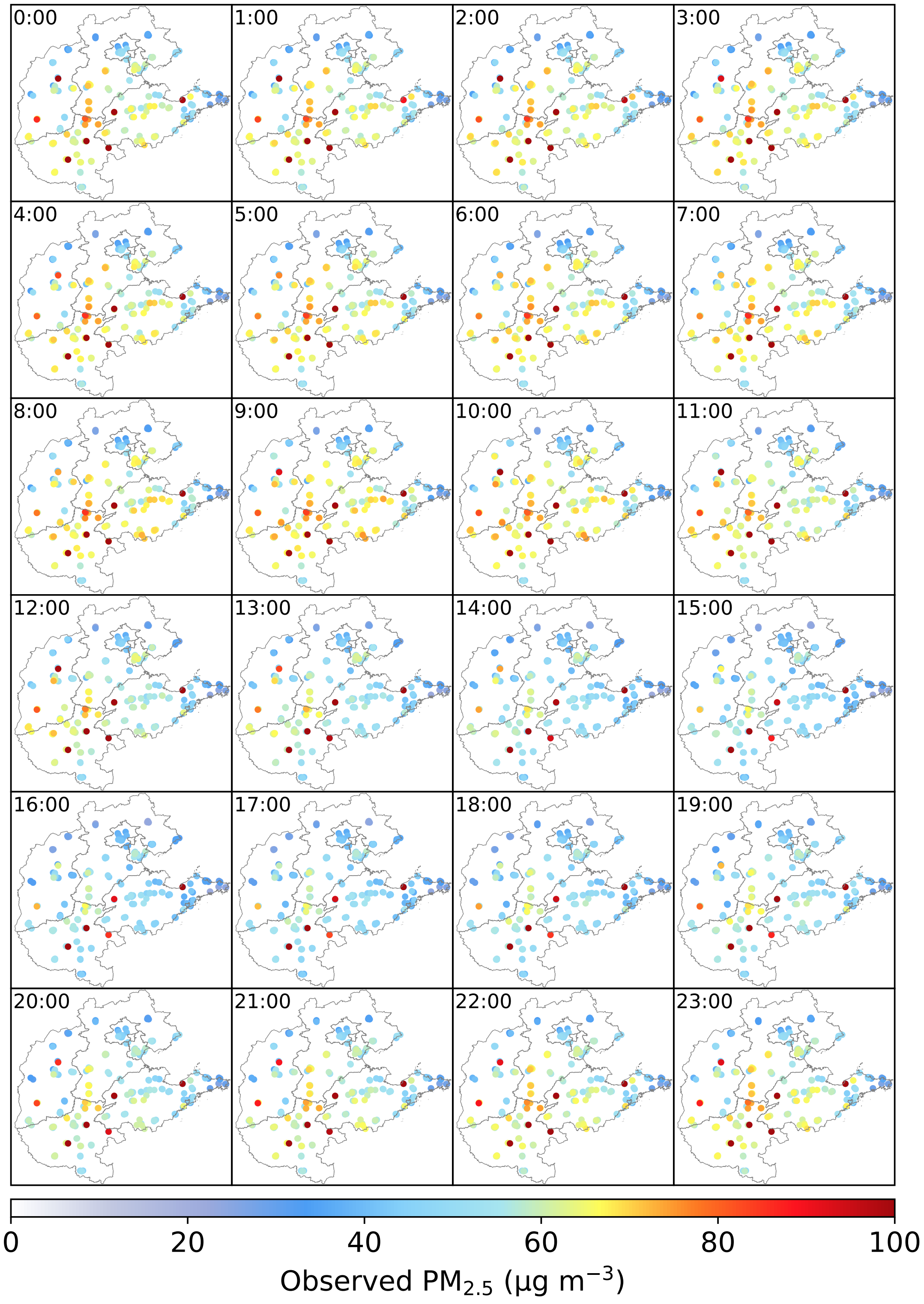

Fig. S10 Diurnal variations in the spatial distribution of observed $PM_{2.5}$ in 2019 across the North China Plain.

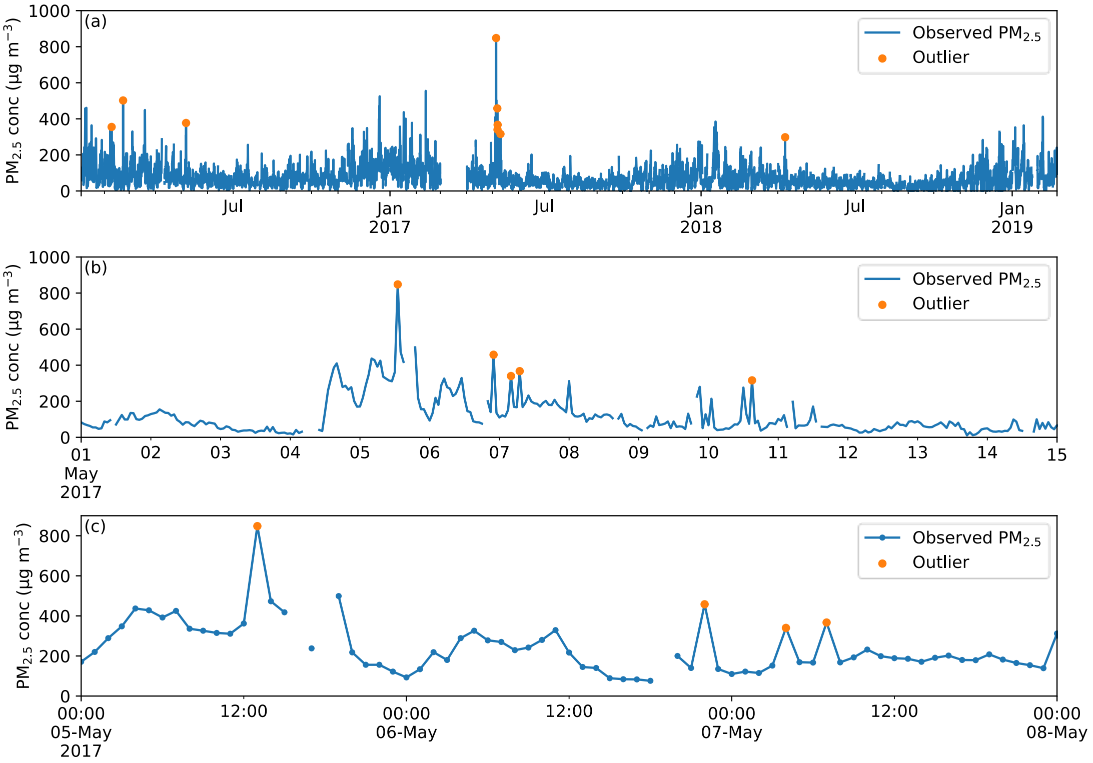

Fig. S11 Time series of hourly $PM_{2.5}$ observations at one station from 2016 to 2019 to show the effect of method for identifying and filtering outliers (b is the local zoom of a, c is the local zoom of b).
